# Successful Operation for the Radical Cure of Hernia

**Published:** 1861-04

**Authors:** Richard McSherry

**Affiliations:** Baltimore


*Successful Operation for the Radical Cure of Hernia.* By RICHARD MCSHERRY, M.D., of Baltimore.

Mr. P., a fine youth of seventeen years of age, applied to me on the 28th of September last, to know whether he could be cured of an oblique scrotal hernia, on the right side, which had been troubling him for the last three years. He had been a pupil at a military school, and was very active in his habits of life, was fond of field sports, and had been much in the saddle. He was greatly distressed at having a disease which interfered so materially with his comfort and prospects, and he was quite ready to adopt any course likely to effect a permanent cure.

I believed that this could be accomplished in his case by Wutzer's operation, which I explained to him, when he consented at once to submit to it. On the 1st of October, then, preparation was made for the operation by clearing out the bowels freely with castor oil, and by removing the capilli, which he partially plucked away with tweezers, but finding the process tedious the parts were shaven by a razor so far as necessary.

On the 2d of October, assisted by my friend, Dr. W. C. Van Bibber, I performed the operation of applying Wutzer's instrument in the usual manner, which will now be described, with all the leading details of the case. The instrument, it should be stated in the first place, is not of the cylindrical form used by Wutzer, but it is flattened (one's finger, or thumb, firmly compressed upon a resisting surface gives a good idea of the form) after the suggestion of Mr. Spencer Wells, the transverse diameter being much greater than the antero-posterior. This modification is a practical improvement on the cylinder, inasmuch as it converts the ring into the form of a chink, offers additional security against the descent of the intestine and leaves the invaginated surfaces more approximated to each other when the instrument is removed.

The patient being placed on his back on the side of his bed, thigh slightly flexed and shoulders slightly elevated, after Dr. Van Bibber and myself had carefully traced the canal, which easily admitted the forefinger up to the internal ring, I inserted the instrument, well greased, up to the orifice, withdrawing at the same time the finger which I had used as a guide. The wooden plug, with the invaginated integuments, being *in situ*, I passed the needle (about the size of a crow quill, with a trocer point and plated surface, except, of course, at the point,) along the tube in the plug, and through the invaginated and external i ntegnments and intermediate tissues. The wooden compress was then applied with moderate firmness, the point was capped, and the operation was complete. The patient complained smartly of pain. I left him to return at the end of two hours. Upon my return I found him complaining still more of the severity of the pain, which, he said, was most severe where the plug pressed against the falling bowel—that is, at the internal ring. I eased the pressure by loosening the screws, which gave a little relief; but he found still more from having a firm pillow placed under his hips, so as to elevate the lower portion of the pelvis. I gave him, moreover, thirty drops of the elixir of opium, which was repeated at bedtime.

The next morning I found him very much more comfortable, free from severe suffering, but complaining of numbness along the thigh, owing to pressure on the anterior crural nerve. There was considerable tenderness to the touch, the soreness radiating from the point punctured. The tenderness was most decided above the internal ring, and in a line towards the anterior superior spinous process of the ilium. Coughing or sneezing always gave painful impulse.

On the 4th, 5th and 6th suppuration was gradually developed around the puncture, the process of which could readily be observed through the slit in the wooden compressor.—There were no unpleasant symptoms; surrounding soreness was soothed by the application of olive oil and laudanum.

On the 8th he complained of unusual pain, principally in the upper portion of the thigh below the groin. There was considerable soreness along the course of the canal in which the instrument lay. There was no fever, but he slept badly and was becoming nervous. To allay excitement, I ordered thirty drops of the elix. opii to be given at bed-time, which produced a good effect.

On the morning of the 10th I removed the instrument, there being at the time considerable pain in the surrounding parts, with nervous and febrile excitement. The general condition of things was quite satisfactory when the instrument was removed. Some sero-purulent matter escaped from the outlet of the invaginated skin, or *doigt de grant*, as it is sometimes appropriately called. I applied a suspensory bandage only, directing him to keep at rest, and to cleanse the parts with soap and tepid water. During all the time he had been upon very light diet, and there had been no action on the bowels. On the 12th a dose of oil cleared out the intestinal canal; before it was taken I applied a hard compress over the tract of invagination and bound it down with adhesive strips.

From this time there was a steady improvement day by day. By the 20th the puncture was quite well, and all tenderness to pressure was gone. By the 27th he was able to walk out, wearing a truss and suspensory bandage by way of precaution. The outlet of the invagination exhibited the moisture of mucus membrane.

He returned to school early in December. On the 24th he called to see me; was perfectly well; wore his suspensory bandage, but no truss, which he objected to on account of some induration and sensibility of the spermatic cord. He had been pursuing his usual occupations without any hindrance, had been gunning, and riding on horseback, though I had advised him to defer such trials for some time longer. The invaginated canal was quite closed, and the orifice dry, there being no longer any exudation.

I had the pleasure of hearing this morning (January 31st) that he continues perfectly well.

It would be superfluous to make any extended remarks on this case, or on the operation. My only object is to record an additional instance of radical cure, and not to discuss the merits or demerits of the operation. That many failures have followed upon it is very certain; that it has been attended with many cures is equally certain. There is scarcely anything in surgical practice more important than the radical cure of hernia, and any means of effecting this, not involving, under any ordinary circumstances, danger to life, is exceedingly desirable. Wutzer's operation has been more successful than any other so far devised by surgical skill. Under circumstances perfectly favorable, as in the case which I have here recorded, there is every reason to hope for success from it. Failures may be expected to follow in bad cases, in operations ill performed, or from the use of defective instruments. But the principle involved is good, and the surgeon must be ready to modify his operations according to the requirements of his cases. In large and old ruptures, the operation I performed upon this young gentleman would be inadequate; but it may be suitably modified. Prof. N. R. Smith, of this city, suggests in such bad cases the use of a large plug with several metallic pins, or tacks, inserted at different points from without. This would require that the plug should be of soft wood, that the pins, which might be made of steel, gilt or plated, with screw-heads, could be readily inserted into it. The suggestion is certainly a good one. Perhaps nothing would be better than metallic, or even thread, sutures, not entering the wood at all, which would act as fine setons.

So far as Wutzer's operation is concerned, the surgeon may deviate according to his own judgment, from the manner of it, or from the original instrument, so that he keeps the principle in view; which is to plug up a patulous orifice and canal with a new growth sufficiently firm to resist the usual pressure from within the abdominal cavity. By adapting the operation in every case to the existing exigencies, there is no doubt, in my mind, that the result will be, most commonly, successful.—*Md. & Va. Med. Journal.*

